# Assessment of Retrospective COVID-19 Spatial Clusters with Respect to Demographic Factors: Case Study of Kansas City, Missouri, United States

**DOI:** 10.3390/ijerph182111496

**Published:** 2021-11-01

**Authors:** Hadeel AlQadi, Majid Bani-Yaghoub, Sindhu Balakumar, Siqi Wu, Alex Francisco

**Affiliations:** 1 Department of Mathematics and Statistics, University of Missouri-Kansas City, Kansas City, MO 64110, USA; baniyaghoubm@umkc.edu (M.B.-Y.); sbmbg@mail.umkc.edu (S.B.); sw4fc@mail.umkc.edu (S.W.); 2 Department of Mathematics, Jazan University, Jazan 45142, Saudi Arabia; 3 City of Kansas City Health Department, 2400 Troost Ave, Kansas City, MO 64108, USA; Alex.Francisco@kcmo.org

**Keywords:** disease surveillance, gender, race, ethnicity, COVID-19, SatScan, cluster analysis, spatial analysis, pandemic, time series

## Abstract

Coronavirus disease 2019 (COVID-19) is caused by severe acute respiratory syndrome coronavirus 2 (SARS-CoV-2). The United States (U.S.) has the highest number of reported COVID-19 infections and related deaths in the world, accounting for 17.8% of total global confirmed cases as of August 2021. As COVID-19 spread throughout communities across the U.S., it became clear that inequities would arise among differing demographics. Several researchers have suggested that certain racial and ethnic minority groups may have been disproportionately impacted by the spread of COVID-19. In the present study, we used the daily data of COVID-19 cases in Kansas City, Missouri, to observe differences in COVID-19 clusters with respect to gender, race, and ethnicity. Specifically, we utilized a retrospective Poisson spatial scan statistic with respect to demographic factors to detect daily clusters of COVID-19 in Kansas City at the zip code level from March to November 2020. Our statistical results indicated that clusters of the male population were more widely scattered than clusters of the female population. Clusters of the Hispanic population had the highest prevalence and were also more widely scattered. This demographic cluster analysis can provide guidance for reducing the social inequalities associated with the COVID-19 pandemic. Moreover, applying stronger preventive and control measures to emerging clusters can reduce the likelihood of another epidemic wave of infection.

## 1. Introduction

Coronavirus disease 2019 (COVID-19) is caused by severe acute respiratory syndrome coronavirus 2 (SARS-CoV-2), and is transmitted via respiratory droplets and direct contact [1,2] Symptoms, which may include fever, chills, cough, or shortness of breath, appear 2–14 days after exposure to the virus [3]. The case fatality rate of COVID-19 is approximately 2% to 3%, but it is highly contagious and has developed rapidly worldwide [2].

The first COVID-19 case in the United States was reported on 21 January 2020 [4]. The World Health Organization [5] declared this outbreak a pandemic in March 2020. As of August 2021, the U.S. has the highest number of reported COVID-19 infections and related deaths in the world, accounting for 17.8% of total global confirmed cases. Among 3142 U.S. counties, 247 counties reached the 90th percentile for hospitalization-per-bed, and 136 counties were in the 90th percentile for ICU-per-bed [6].

Stricter lockdown measures were enforced to combat rising COVID cases, resulting in a global economic crisis. In the U.S., unemployment rates increased to 14.7% in April 2020 [7]. The pandemic caused economic disruptions and an exceptional increase in uncertainty in the U.S. economy, which will likely drive the U.S. economy into recession [8]. While following the CDC guidelines, each county and major U.S. city developed its own timing, range, breadth, and depth of actions depending on geographic and demographic differences and political diversity [9].

As COVID-19 spread throughout communities across the U.S., it became clear that inequities would arise among differing demographics. Several studies suggested that certain racial and ethnic minority groups may have been disproportionately impacted by the spread of COVID-19 [10,11]. For instance, the African American population comprises 13% of the total U.S. population [12], while according to CDC COVID-19 data [13], it made up 30% of all COVID-19 positive cases in the U.S. as of 15 April 2020. In addition, hospitalization rates due to COVID-19 have a disproportionately higher prevalence among the African American population than among any other race [14].

In addition, Hispanics are currently the largest ethnic minority in the U.S., with a population of nearly 60 million people [15]. While Hispanics account for 18% of the total U.S. population, they constitute nearly 28.4% of the total COVID-19 cases nationally [15]. Nevertheless, the proportions vary strongly in different parts of the country. For instance, a statewide study investigating racial disparities followed 3481 COVID-19 cases in Louisiana and found that non-Hispanic African American individuals represented 77% of hospitalizations and 71% of deaths, despite only making up 31% of the total source population [10,11]. In New York City, COVID-19 mortality rates were 220 and 236 per 100,000 for African American and Latinx patients, respectively, whereas White and Asian Americans had mortalities of 110 and 102 per 100,000, respectively [11].

Of particular interest, the City of Kansas City, MO, issued a state of emergency proclamation on 12 March 2020, which effectively placed limits on gatherings and mandated masks [16]. Like other U.S. cities, social distancing measures were applied throughout Kansas City, MO. Kansas City, MO became one of the U.S.’s COVID-19 hot spots due to a substantial increase in the rate of positive COVID-19 test results in 2020. It also became one of 10 cities across the U.S. that received the attention of the White House coronavirus task force in 2020 [17]. Despite efforts to reduce the prevalence of infection, 40,486 confirmed cases and 597 deaths had been reported in Kansas City as of 8 July 2021 [18]. With the increase in COVID-19 vaccinations, and in accordance with CDC guidelines, on 14 May 2021, Kansas City ended its emergency order requiring masking and social distancing. Despite the rise in vaccinations, cases have continued to increase, highlighting the need for a better understanding of how COVID-19 is spreading across the city.

As of 21 October 2021, the World Health Organization (WHO) database shows that out of 310,548 papers related to COVID-19, there are only 37 papers focusing on Kansas City. Most of those papers are associated with medical aspects of COVID-19 in Kansas City, MO [19,20,21,22,23]. The other papers relate to factors such as mask mandates [24,25], vaccine aspects and patient hospitalization [24,26]. One of these papers involved a retrospective study on COVID-19 data examining gender and race [19]. However, the study focused on the therapeutic factors that influenced COVID-19 patients’ ICU admissions with respect to gender and race. Another study analyzed data from both St. Louis and Kansas City, MO to compare COVID-19 testing rates per diagnosed cases between races [27]. By studying COVID-19 testing data over time, this study concluded that black populations observed lower COVID-19 testing rates than white populations. Another paper focused on COVID-19 cases in relation to income level by studying specific zip codes in Kansas City, MO [28]. One main finding was that an increase in median income level was associated with a decrease in the health risk gap. The above-mentioned papers contribute to the existing literature. However, they largely ignore how the geographical distributions of COVID-19 cases may be impacted by demographic factors.

It is crucial to establish spatial monitoring during an evolving pandemic with several strains, such as COVID-19. Spatial statistical clustering can be used as a COVID-19 surveillance tool in communities. Among the global measures of spatial clustering analysis, spatial scan statistics is one of the most popular and powerful techniques applied to perform geographical surveillance of diseases [29]. Spatial cluster analysis has been used in a wide variety of epidemiological studies to evaluate spatial patterns of infections [30,31,32]. Some examples include identifying birth defects in New York State [33] and studying spatial patterns of malaria and detecting spatial clusters of tuberculosis in China [32,34]. In fact, spatial clusters can be more valuable in identifying regions with an increased risk of COVID-19 in certain racial and ethnic minority groups if we consider the demographic factors. Cluster detection of when and where COVID-19 transmission occurs is critical to preventing another wave, preventing small local outbreaks in certain communities, and eventually controlling the epidemic by establishing testing and vaccination centers in the most impacted regions. To our knowledge, there is no published research article that retrospectively examines COVID-19 spatial clusters with respect to demographic factors in Kansas City, MO. Hence, the main purpose of the present study was to provide an unbiased spatial and temporal analysis of COVID-19 data with respect to demographic factors in Kansas City, MO. Using the COVID-19 data, we analyzed the progression of COVID-19 infection in Kansas City with respect to gender, race, and ethnicity. This study implements a retrospective spatial analysis of COVID-19 in Kansas City, MO, at the zip code level for the period March–November 2020.

## 2. Materials and Methods

### 2.1. Study Area

**Figure 1:**
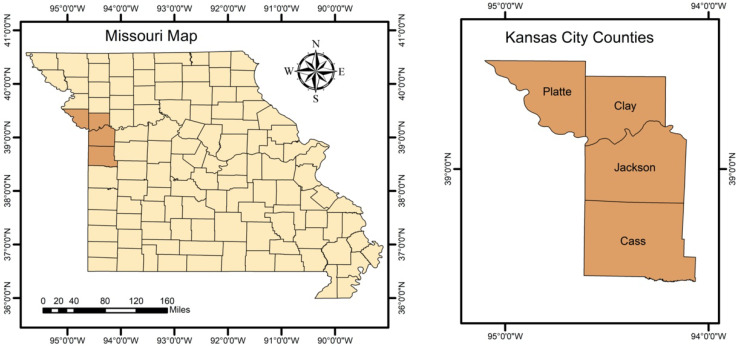
Missouri state map (**left**), Kansas City counties map (**right**).

The study area is Kansas City, MO. Kansas City lies within Jackson County, with some portions spilling into Clay, Cass, and Platte counties between latitude 39°05′59″ N and longitude 94°34′42″ W (Figure 1). The space-time analysis was conducted in all four Kansas City counties. Based on the U.S. Census Bureau American Community Survey, Kansas City, MO had an estimated population of 508,090 in 2020. Approximately 51.5% of the population was female, 60.9% was White, and 23.1% of the total population was under 18 years old [12].

### 2.2. Data Source

The City of Kansas City Health Department (KCMO Health Department) provided daily confirmed cases of COVID-19 between March and November 2020. This study was approved by the KCMO Health Department. It was granted a waiver of informed consent and is compliant with the Health Insurance Portability and Accountability Act.

The case data contain the following variables: date of case receipt, Epidemiological (Epi) week, Epi year, EpiTrax CMR# [35], age, gender, race, zip code, specimen collection date, vital status, and outbreak associated. In addition, we collected population data that contained the background of each demographic factor and coordinate data that contained latitude and longitude for each zip code [36]. Table 1 shows the summary statistics of the number of COVID-positive cases and mortality rates in Kansas City from March to November 2020.

**Figure 2:**
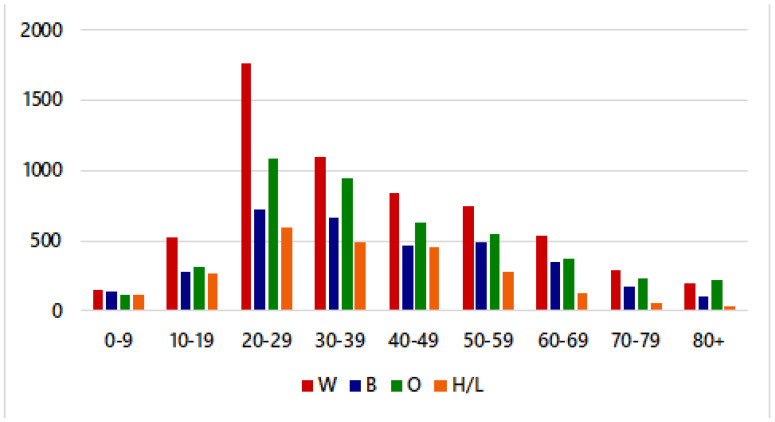
Crude COVID-19 cases by age & race/ethnicity in Kansas City, MO, from March to November 2020.

Figure 2 shows that individuals aged 20–29 years had the highest number of confirmed cases in Kansas City, MO. Moreover, White individuals aged 20–29 years had the highest number of confirmed cases. Note that to access potential racial disparities in the spread of COVID-19, it is important to consider the proportion of confirmed cases in Kansas City, MO with respect to race/ethnicity. Figure 2 shows the raw data for individuals who are White (W), African American (B), Other (O), and Hispanic/Latino (H/L).

The “Other” category comprises people who identify as American Indian, Hawaiian/Pacific Islander, or Asian. In the raw data, White individuals had the greatest proportion of COVID-19 cases (34.82%), while Hispanic/Latino individuals had the lowest proportion of cases (13.76%). Note that the proportion of cases does not reflect the prevalence of cases by race/ethnicity.

### 2.3. Methods

In this paper, we aimed to observe the differences in COVID-19 cases with respect to demographic factors. Hence, we utilized a one-way analysis of variance (ANOVA) test to observe if there were any statistically significant differences in COVID-19 positive cases in terms of demographic factors. In addition, we conducted retrospective spatial scan statistics to detect significant spatial COVID-19 clusters with respect to demographic factors. For the explanation of the process of the selected statistical methods, see Figure S1 in the Supplementary Materials.

#### 2.3.1. Statistical Analysis

We utilized a one-way ANOVA to determine whether any statistically significant differences existed between the COVID-19 positive cases with respect to the average ages of White, Black, and Hispanic subpopulations. Hence, the null and alternative hypotheses were as follows:

**Hypothesis 1**. *The mean values of COVID-19 cases are the same with respect to race and ethnicity.*

**Hypothesis 2**. *There is at least one mean value that is different from the others.*

The null hypothesis was that the mean values are the same with respect to each race and ethnicity. The alternative hypothesis stated that there is at least one mean value which is different from the other two. We could reject the null hypothesis and accept the alternative hypothesis if the *p*-value was less than 0.05. Next, a post-hoc test was used to determine whether a statistically significant difference existed between each group. A paired-samples *t*-test was used assuming equal variances compared to each relationship among the three groups.

#### 2.3.2. Cluster Analysis

We were interested to detect significant spatial COVID-19 clusters at the zip code level in Kansas City with respect to demographics (i.e., gender, race, and ethnicity). The analysis would help us understand how the geographical distribution of COVID-19 cases associated with demographic factors.

To analyze the spatial clusters of COVID-19 with respect to gender, race, and ethnicity, a purely spatial analysis was conducted using the spatial scan statistic of the discrete Poisson model implemented in the SaTScan software [37].

Briefly, we assumed that COVID-19 cases followed the Poisson model because we were interested in the geographical distribution of the COVID-19 cases associated with demographic factors, adjusting for each population at risk. The retrospective space analysis used a circular window that moved in one dimension, using the base of the cylindrical window as the space dimension. The maximum cluster size was set to 25% of the population at risk to avoid extremely large clusters. Thus, numerous overlapping windows of different sizes were generated, which together covered the entire study area. Each circular window was considered a possible candidate cluster. Hence, the null and alternative hypotheses were as follows:

**Hypothesis 3**. *The disease risk remains the same inside and outside the scanning window.*

**Hypothesis 4**. *The risk within the window is different from that outside the window.*

Each cylinder was expanded until a maximum cluster size upper bound was reached. A likelihood ratio test was used to identify the spatial clusters of COVID-19 cases [38]. The likelihood ratio was defined by the following formula:

$$\frac{L\left(C\right)}{L_{0}}=\frac{{\left(\frac{n_{c}}{\mu \left(c\right)}\right)}^{n_{c}}{\left(\frac{N-n_{c}}{N-\mu \left(c\right)}\right)}^{N-n_{c}}}{{\left(\frac{N}{\mu \left(T\right)}\right)}^{N}}.$$

Namely, the likelihood ratio was calculated based on the observed and expected number of cases inside and outside the circle C, where is *L(C)* is the maximum likelihood function for the cylinder with base C, *L_0_* is the likelihood function under the null hypothesis, *n_c_* is the number of COVID-19-positive cases for each demographic factor in the cylinder, *µ(c)* is the number of expected cases for each demographic factor in the cylinder, *N* is the total number of all observed cases in Kansas City over the time interval with respect to gender, race and ethnicity, and *µ(T)* is the total number of expected cases in Kansas City between March and November 2020 with respect to gender, race and ethnicity. The likelihood was calculated for each cylinder to determine whether the observed number of cases exceeded the expected number of cases (i.e., if the likelihood ratio was greater than 1). The window with the maximum likelihood ratio statistic constituted the likeliest cluster (primary cluster). Secondary clusters were also reported if they were statistically significant at the *p*-value < α = 0.05. The *p*-values for space-time clusters were estimated using Monte Carlo simulations by repeating *n* = 999 random iterations of the dataset to test the significance [39].

The results section provides the statistically significant emerging clusters of COVID-19 in Kansas City, MO, at the zip code level with respect to demographic factors: gender, race, and ethnicity. The spatial clusters were analyzed using SaTScan^TM^ 9.6, and the maps were plotted using ArcGIS 10.8.

## 3. Results

### 3.1. Descriptive Statistics

**Figure 3:**
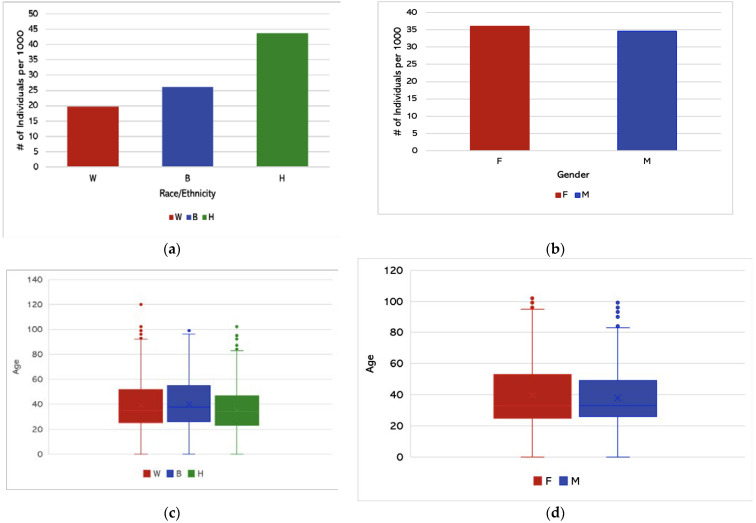
(**a**) Prevalence of COVID-19 cases in Kansas City with respect to race and ethnicity from March to November 2020. (**b**) Prevalence of COVID-19 cases with respect to gender from March to November 2020. (**c**) Average age of COVID-19 cases by race/ethnicity (per 1000 individuals) in Kansas City. (**d**) Average age of COVID-19 cases by gender (per 1000 individuals).

We obtained the descriptive statistics involving race/ethnicity and age distribution using Microsoft Excel. Figure 3a shows the prevalence of COVID-19 cases with respect to specific race/ethnicity groups. Hispanic/Latino individuals had the highest prevalence of COVID-19 cases in Kansas City, while White individuals had the lowest prevalence. Figure 3b shows the prevalence of COVID-19 cases with respect to gender. Figure 3c shows that the average age of White individuals testing positive for COVID-19 was 39.1 years, the average age of African American individuals was 40.3 years, and the average age of Hispanic individuals was 35.7 years.

Prevalence was calculated by dividing the number of cases within a specific racial group by the total number of individuals belonging to that same racial group in Kansas City, MO, and multiplying that decimal by 1000 to obtain the prevalence per 1000 individuals. Figure 3a,b represents the prevalence of COVID-19 positive cases per 1000 with respect to race/ethnicity and gender. It was observed that Hispanics had higher prevalence than White and African American groups. Figure 3c,d represent the average age of COVID-19 cases with respect to race/ethnicity and gender, respectively.

### 3.2. Hypothesis Testing

We utilized a one-way ANOVA to determine whether statistically significant differences existed between the average ages of the White, African American, and Hispanic groups.

Table 2 shows the results of a one-way ANOVA test. Given that the *p*-value was less than 0.05, there was strong evidence against the null hypothesis and, thus, we accepted the alternative hypothesis that there was at least one average age different from the others. Next, we used a post-hoc test to determine whether a statistically significant difference existed between each group. We found the two-tailed *p*-value of each comparison and compared that to the Bonferroni correction value of 0.0167. The average age of White individuals testing positive for COVID-19 was 39.1 years, the average age of African American individuals was 40.3 years, and the average age of Hispanic individuals was 35.7 years. From Table 3, we can see that each *p*-value was less than the Bonferroni correction value of 0.0167, which means that the average age of Hispanic individuals was significantly lower than that of both African American and White individuals. Hence, the average age of COVID-positive individuals was significantly different with respect to every race/ethnicity.

### 3.3. Times-Series Analysis

**Figure 4:**
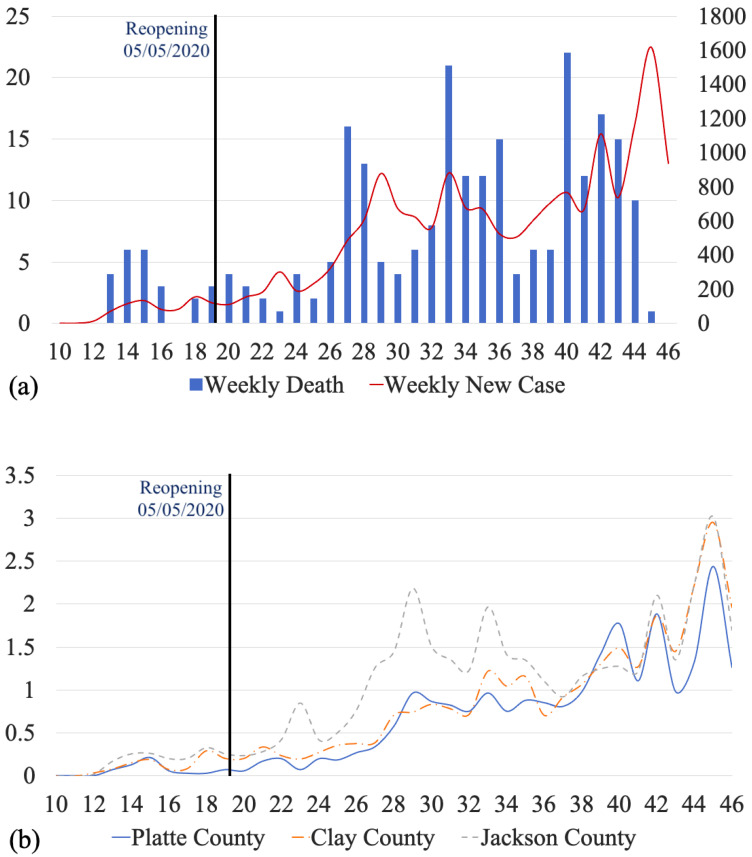

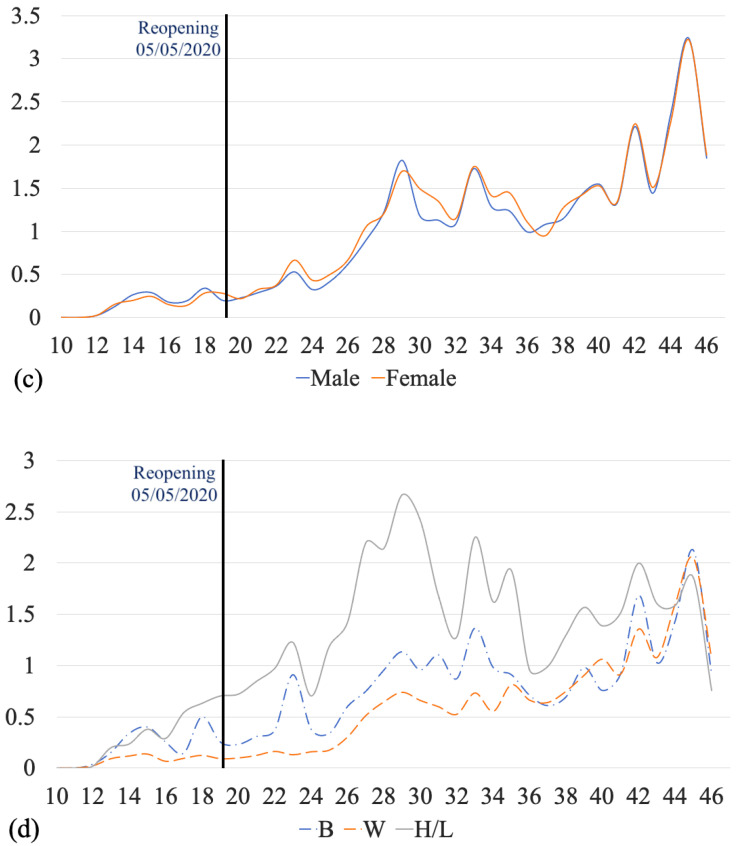
Time series of COVID-19 cases in Kansas City, MO, during March–November 2020. (**a**) Number of weekly new cases and mortality, (**b**) number of weekly new cases per thousand by county, (**c**) number of weekly new cases per thousand by gender, and (**d**) number of weekly new cases per thousand by race and ethnicity.

We monitored the temporal variations in the number of COVID-19 cases between March and November 2020. Note that there were 545 cases from zip codes that did not belong to Kansas City, MO, or could not be found on the map. There were also 43 cases with “unknown” zip codes. Therefore, these 588 cases were eliminated from the original data for Figure 4b. For the population of each county based on the zip codes, see Tables S1–S4 in the Supplementary Document.

Figure 4a shows weekly COVID new cases and mortalities in Kansas City, MO, during March–November 2020. The total number of cases during March–November 2020 was 17,650, where 648 cases occurred before reopening (5 May 2020), and the rest (17,003) after reopening. The time series of the cases (shown with a red curve) has multiple M-shaped (double-top) curves. There is a small “double-top” from the 12th week to the 20th week with the maximum values in the 15th and 18th weeks, and the minimum in the 16th week. There are also two “double-tops” with larger magnitudes, one from the 27th week to the 37th week and the other from the 41st week to the 46th week. Figure 4b shows the graph of weekly cases per thousand for Clay County, Platte County, and Jackson County. Before the 16th week, all counties had similar trends of cases per thousand, but Jackson County had more cases per thousand than Clay County and Platte County. Between the 22nd week and the 37th week, Jackson County had more cases per thousand. Between the 39th week and the 40th week, Platte County had more cases per thousand. From the 41st week, all counties had the same trend of cases per thousand, but Platte County had fewer cases per thousand than Clay County and Jackson County. Figure 4c shows weekly cases per thousand by gender in Kansas City. Males and females had similar trends and similar rates of cases. Figure 4d shows weekly cases per thousand by race in Kansas City. The three races are White, African American, and Hispanic/Latino. Hispanic/Latino always had the highest rate of cases. Before the 36th week, African-American individuals had higher rates than White individuals. After the 36th week, African American and White individuals had similar trends and rates of cases. From week 44 to week 46, the three races had a close trend and rate of cases.

### 3.4. Cluster Analysis

The results of the cluster analysis are divided into two parts: spatial clusters of COVID-19 with respect to gender, and spatial clusters of COVID-19 with respect to race and ethnicity.

**Figure 5:**
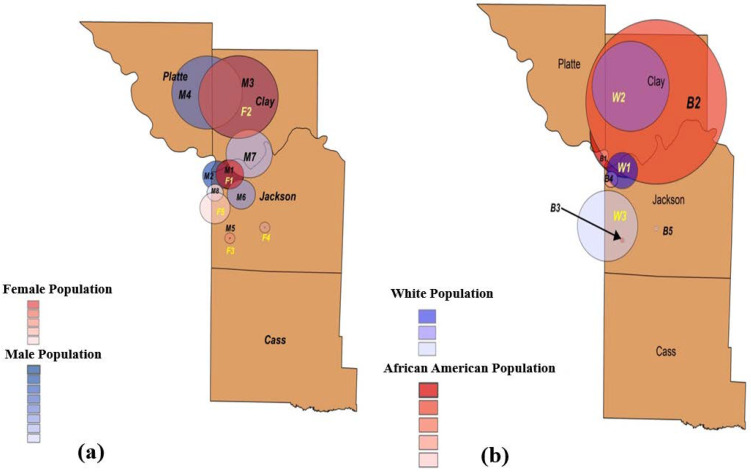

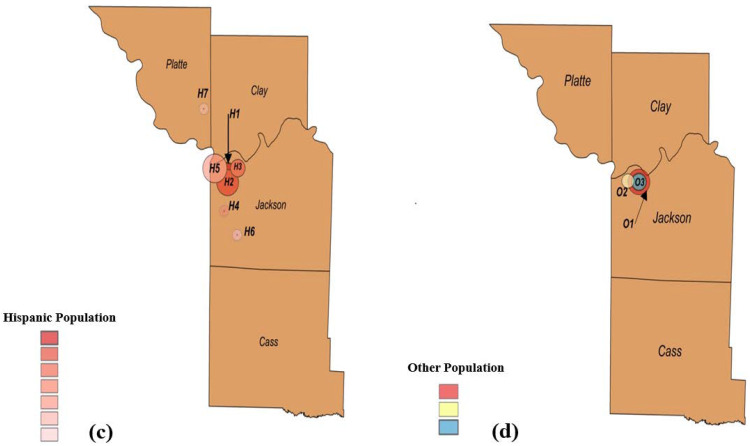
Emerging spatial clusters of COVID-19 with respect to demographic factors. Spatial clusters with respect to (**a**) gender, (**b**) White and African American populations, (**c**) Hispanic population, and (**d**) other races, i.e., Asian, American Indian, and Hawaiian and Pacific Islander.

Figure 5a and Table 4 provide the characteristics of the spatial clusters of COVID-19 with respect to gender in Kansas City at the zip code level from March to November 2020. We observed five statistically significant clusters of females and eight statistically significant clusters of males. The relative risks (RRs) of the primary clusters F1 (female) and M1 (male) were almost the same, and both clusters were in downtown Kansas City, located in Jackson County. As we can see in Figure 5a, the clusters of the male population were more scattered than those of the female population. In addition, note that the male and female populations share three clusters.

Figure 5b and Table 5 provide details of the spatial COVID-19 clusters with respect to White and African American populations in Kansas City at the zip code level from March to November 2020. There were five spatial clusters belonging to the African American and three spatial clusters belonging to the White population. Note that the RR for the primary cluster (W1) of the White population was almost twice that of the RR for the primary cluster (B1) of the African American population. However, cluster B5 had a greater RR than that of primary cluster B1. Figure 5b shows that the clusters of the African American and White populations were mainly shared in Clay County. Note that the secondary cluster B2 of the African American population covered almost the entire county.

Figure 5c and Table 6 show the spatial clusters of COVID-19 with respect to race and ethnicity for Hispanic population in Kansas City at the zip code level from March to November 2020. There were seven spatial clusters of Hispanic population. These clusters had the highest prevalence compared to those of the African American and White populations, and they were scattered mainly in Jackson County. Cluster H4 of the Hispanic population had an extremely large RR of 4.77. Figure 5d and Table 6 show the clusters that belong to other races, including Asian, American Indian, and Hawaiian and Pacific Islander populations. The spatial clusters for these races were located in Jackson County, specifically downtown Kansas City.

## 4. Discussion

Spatial cluster analysis is often utilized to identify regions with an increased risk of occurrence of studied phenomena. Studying the cluster patterns of COVID-19 can provide evidence for specific risk factors that influence the spread of this virus, establishing improved prevention and control measures. Understanding the spatial patterns can also enable public health departments and decision-makers to implement more COVID-19 vaccination clinics in high-risk areas, controlling the severity and spread of disease. Thus, the results of this study can assist the KCMO Health Department in developing more effective prevention strategies in future.

The main purpose of the present study was to provide an unbiased spatial and temporal analysis of COVID-19 data in Kansas City, MO. We examined significant differences in COVID-19 clusters with respect to demographic factors of gender, race, and ethnicity. Our research is the first study to utilize retrospective spatial statistics adjusted for demographic factors to monitor COVID-19 in Kansas City, MO.

Retrospective spatial analysis was able to identify eight clusters belonging to male populations, five spatial clusters for African American populations, and seven spatial clusters representing Hispanic populations, which had the most scattered clusters and the highest prevalence of COVID-19 cases when compared to those of the African American and White populations. Most of the spatial demographic clusters concentrated in downtown Kansas City, located in Jackson County. In addition, the average age of Hispanic individuals with COVID-19 was significantly lower than that of both African American and White individuals. This finding was identified via ANOVA testing which determined a statistically significant difference between the three groups (Hispanic, Black, and White individuals), followed by a post-hoc test which determined the differences in the means.

We also monitored the temporal variations in the number of COVID-19 cases between March and November 2020. The time series of the cases had multiple M-shaped (double-top) curves. There were three obvious M-shaped curves that showed the increase in cases occurring with a type of period. Five weeks after reopening, a continuous increase appeared for four weeks, followed by the appearance of an M-shaped curve. Reopening influenced weekly new COVID-19 cases in the short term. In addition, compared to the time series of weekly cases per thousand for Clay County and Platte County, Jackson County had the greatest number of cases per thousand for the first 24 weeks. However, after the 37th week, the COVID-19 outbreak shifted. The trend and number of cases per thousand for the three counties became similar. The time series of Hispanic/Latino cases led the temporal trend until the 44th week.

Despite the contributions of our study, there are several limitations and assumptions. First, to understand why certain communities have been severely impacted by this pandemic, it is important to study the health inequities and social determinants of health that contribute to current conditions. Certain factors such as socioeconomic status, medical conditions, healthcare access, and barriers, such as language and immigration status, may impact the spread of COVID-19 across regions. For instance, cluster H4 of the Hispanic population had an extremely large RR of 4.77. The region of this cluster covered three blocks east of Highway 71 and north of E. 75th Street. Of 361 Hispanic individuals in this region, 61 (16.8%) tested positive for COVID-19. Therefore, it is important to study the factors that impact the spread of COVID-19 in such areas. Being uninsured is another factor that may prevent individuals from seeking proper COVID-19 testing and treatments. In Kansas City, MO, the age-adjusted death rate for Hispanics was 2.7 times higher than for White residents, and the age-adjusted death rate for African Americans was 1.6 times higher than for White residents. Pre-existing medical conditions and access to healthcare may have contributed to the disproportionate impact of COVID-19 on the Hispanic community. Moreover, some researchers suggested that COVID-19 cases in Kansas City, MO were related to income level [28]. The health risk gap narrows as the median income rises, and being white and having a higher median income offers a significant advantage compared to racial and ethnic minority groups with lower median incomes [28]. Thus, income level may contribute to the disproportionate impact of COVID-19 on certain communities. Second, only confirmed cases were included in the KCMO Health Department data, and it is vital to note that unconfirmed and probable cases are not evaluated owing to unavailability and uncertainty. As a result, a full perspective on the impact of COVID-19 in Kansas City, MO on certain communities will be impossible to obtain for some time. Third, our results focus on data from March–November of 2020, which was prior to vaccine distribution. Therefore, trends, number of emerging clusters, and relative risks would likely be different if we updated the current data. Lastly, COVID-19 is more severe in the elderly [40,41]. Future research can examine spatial clusters with respect to demographic factors that adjust for age and other abovementioned factors (socioeconomic status, income level, and healthcare access).

## 5. Conclusions

Analyzing the daily confirmed COVID-19 cases in Kansas City, MO, at the zip code level enabled us to study the variations in COVID-19 clusters based on gender, race, and ethnicity. From March to November 2020, we used a retrospective Poisson spatial scan statistic to observe the geographical distribution of COVID-19 cases associated with these demographic variables. Our statistical findings revealed that male population clusters were more widely dispersed than female population clusters. Clusters of the Hispanic population were the most prevalent and the most widely dispersed.

Descriptive analysis showed that Hispanic/Latino individuals had the highest prevalence and led the temporal trend of COVID-19 cases in Kansas City when compared with African American and White individuals. Moreover, the average age of Hispanic individuals with COVID-19 was significantly lower than that of both African American and White individuals. When observing the number of new weekly COVID-19 cases by race/ethnicity, we could see that Hispanic/Latino individuals had a higher rate of cases than African American and White individuals.

A spatial and temporal demographic analysis of COVID-19 data enables local health departments to gain a deeper understanding of the regions of Kansas City with increased incidences of cases, as well as the risk factors that influence the spread of disease. Identifying counties and hotspot areas with the highest incidence of COVID-19 cases can help guide Kansas City, MO in deciding where to increase vaccination distribution efforts and COVID-19 testing sites. It may be beneficial to study the areas with statistically significant clusters to ensure that these regions have adequate testing and triage centers within close proximity. Establishing free COVID-19 testing sites and promoting social distancing guidelines, proper hygiene measures, and distributing masks in these areas could help control the spread of COVID-19, as well.

## Figures and Tables

**Table 1:** Summary statistics of the number of COVID-positive cases and mortality rates in Kansas City from March to November 2020.

	Minimum	Maximum	Mean	Median	STD	Range
Cases	13	1618	504.29	524	375.74	1605
Deaths	0	22	7.14	5	6.06	22

**Table 2:** One-way ANOVA to test differences with respect to race and ethnicity.

Group	Count		Sum	Average	Variance	
White	6142		240,691	39.1877	365.1475	
African American	3384		136,436	40.3179	384.2199	
Hispanic	2429		86,656	35.6756	293.8412	
**Source of Variation**	**SS**	**Df**	**MS**	**F**	***p*-Value**	**F Crit**
Between Groups	32,432.19	2	16,216.1	45.5431	0	2.9965
Within Groups	4,255,633	11,952	356.0603			
Total	4,288,065	11,954				

**Table 3:** Post-hoc test with respect to race and ethnicity.

	W vs. B	B vs. H	H vs. W
*P* (T ≤ t) two-tail	0.00637	1.07657 × 10^−20^	3.62 × 10^−15^
Bonferroni correction	0.016667	0.016667	0.016667
*p* < 0.01267	True	True	True

**Table 4:** Spatial clusters of COVID-19 with respect to female (F) and male (M) populations.

Cluster	RR	Observed	Expected	Counties	# of Zip Codes	*p*-Value
Cluster F1	2.21	1907	973.51	Jackson County	9	1 × 10^−17^
Cluster F2	1.41	1421	1052.37	Platte County Clay County	11	1 × 10^−17^
Cluster F3	1.52	253	168.1	Jackson County	1	1.2 × 10^−7^
Cluster F4	2.1	58	27.75	Jackson County	1	6.4 × 10^−5^
Cluster F5	1.11	1913	1757.07	Jackson County	7	4 × 10^−3^
Cluster M1	2.28	1848	926.56	Jackson County	9	1 × 10^−17^
Cluster M2	2.18	1357	682.73	Jackson County	7	1 × 10^−17^
Cluster M3	1.32	1195	937.12	Platte County Clay County	11	2.3 × 10^−15^
Cluster M4	1.34	858	656.17	Platte County	8	1.1 × 10^−12^
Cluster M5	1.56	220	142.81	Jackson County	1	2.8 × 10^−7^
Cluster M6	1.27	472	376.74	Jackson County	3	1.7 × 10^−4^
Cluster M7	1.18	942	810.54	Clay CountyJackson County	7	2.5 × 10^−4^
Cluster M8	1.19	625	530.56	Jackson County	3	3.8 × 10^−3^

Note. The RR for primary clusters F1 (female) and M1 (male) were almost the same, and both clusters were located in downtown Kansas City, Jackson County.

**Table 5:** Spatial clusters of COVID-19 with respect to race and ethnicity: White population (W) and African American population (B).

Cluster	RR	Observed	Expected	Counties	# of Zip Codes	*p*-Value
Cluster W1	3.78	1247	388.13	Jackson County	11	1 × 10^−17^
Cluster W2	1.74	1271	801.10	Clay County Platte County	11	1 × 10^−17^
Cluster W3	1.38	1708	1342.82	Jackson County	13	1 × 10^−17^
Cluster B1	1.89	798	474.39	Clay County Platte County Jackson County	18	1 × 10^−17^
Cluster B2	1.97	681	383.40	Clay County Platte County Jackson County	23	1 × 10^−17^
Cluster B3	1.62	113	70.84	Jackson County	1	2.6 × 10^−4^
Cluster B4	1.64	85	52.45	Jackson County	3	2.4 × 10^−3^
Cluster B5	2.88	18	6.27	Jackson County	1	0.012

Note. The RR for the primary cluster (W1) of the White population was much higher than that (B1) of the African American population. However, cluster B5 had a greater RR than that for the primary cluster B1.

**Table 6:** Spatial clusters of COVID-19 with respect to Hispanic (H) and Other (O) populations (i.e., Asian, American Indian, and Hawaiian and Pacific Islander).

Cluster	RR	Observed	Expected	Counties	# of Zip Codes	*p*-Value
Cluster H1	2.16	985	583.41	Jackson County	5	1 × 10^−17^
Cluster H2	2.17	516	268.92	Jackson County	5	1 × 10^−17^
Cluster H3	1.76	505	315.69	Jackson County	3	1 × 10^−17^
Cluster H4	4.77	61	13.06	Jackson County	1	1 × 10^−17^
Cluster H5	1.69	467	300.60	Jackson County	6	1 × 10^−17^
Cluster H6	1.90	95	50.85	Jackson County	1	2.4 × 10^−6^
Cluster H7	2.39	47	19.86	Platte County	1	1.9 × 10^−5^
Cluster O1	2.61	116	53.83	Jackson County	4	1.1 × 10^−14^
Cluster O2	2.38	57	26.08	Jackson County	1	1.8 × 10^−6^
Cluster O3	2.31	59	27.76	Jackson County	3	2.6 × 10^−6^

Note. The RRs of the primary clusters H1 and O1 were almost the same. However, cluster H4 of the Hispanic population had an extremely large RR.

## Data Availability

The data that support the findings of this study are available on request from KCMO Health Department. The data are not publicly available due to their containing information that could violate HIPAA confidentiality requirements.

## Supplementary Materials

The following are available online at https://www.mdpi.com/article/10.3390/ijerph182111496/s1, Figure S1: Describing the relations of the selected methods. Table S1. Populations of Platte County, Clay County, and Jackson County in Kansas City, Missouri. These populations were collected based on zip code. Table S2. Population of Platte County, Missouri based on the zip codes. Table S3. Population of Clay County, Missouri based on the zip codes. Table S4. Population of Jackson County, Missouri based on the zip codes. Figure S1. Describing the relations of the selected methods.
