# Supplementary material for: Assessment of Retrospective COVID-19 Spatial Clusters with Respect to Demographic Factors: Case Study of Kansas City, Missouri, United States
Source: Int J Environ Res Public Health. 2021 Nov 1;18(21):11496. doi: 10.3390/ijerph182111496 (PMC8582813; doi:10.3390/ijerph182111496)
Supplement: Supplementary file 1 [file ijerph-18-11496-s001.zip › ijerph-1433116-supplementary.pdf]

Supplementary Document

Table S1. Populations of Platte County, Clay County, and Jackson County in Kansas City, Missouri. These populations were collected based on zip code.

**County**	**Platte**	**Clay**	**Jackson**	**total**
**Population**	70,574	155,551	304,558	530,683

Table S2. Population of Platte County, Missouri based on the zip codes.

**zip code**	**population**
64151	26,128
64152	28,307
64153	5,383
64154	10,497
64164	259

Table S3. Population of Clay County, Missouri based on the zip codes.

**zip code**	**population**
64116	15,372
64117	15,001
64118	42,493
64119	26,830
64155	23,108
64156	7,372
64157	19,680
64158	4,796
64165	197
64166	225
64167	477

Table S4. Population of Jackson County, Missouri based on the zip codes.

zip code	Population
64101	279
64102	0
64105	4,306
64106	9,360
64108	7,491
64109	9,188
64110	16,745
64111	17,398
64112	8,698
64113	12,120
64114	24,760
64120	297
64123	9,017
64124	10,534
64125	1,414
64126	6,488
64127	14,707
64128	12,056
64129	8,898
64130	20,215
64131	22,485
64132	14,474
64134	23,836
64136	2,211
64137	11,349
64138	26,485
64139	1,925
64145	5,288
64146	1,574
64147	640
64149	320
64192	0

Populations for each zip code were collected from www.*bestplaces.net*
We used the following resources to find the population of each county based on the zip codes.
Population of Platte County: https://www.bestplaces.net/find/zip.aspx?county=29165&st=MO
Population of Clay County: https://www.bestplaces.net/find/zip.aspx?county=29047&st=MO
Population of Jackson County: https://www.bestplaces.net/find/zip.aspx?county=29095&st=MO

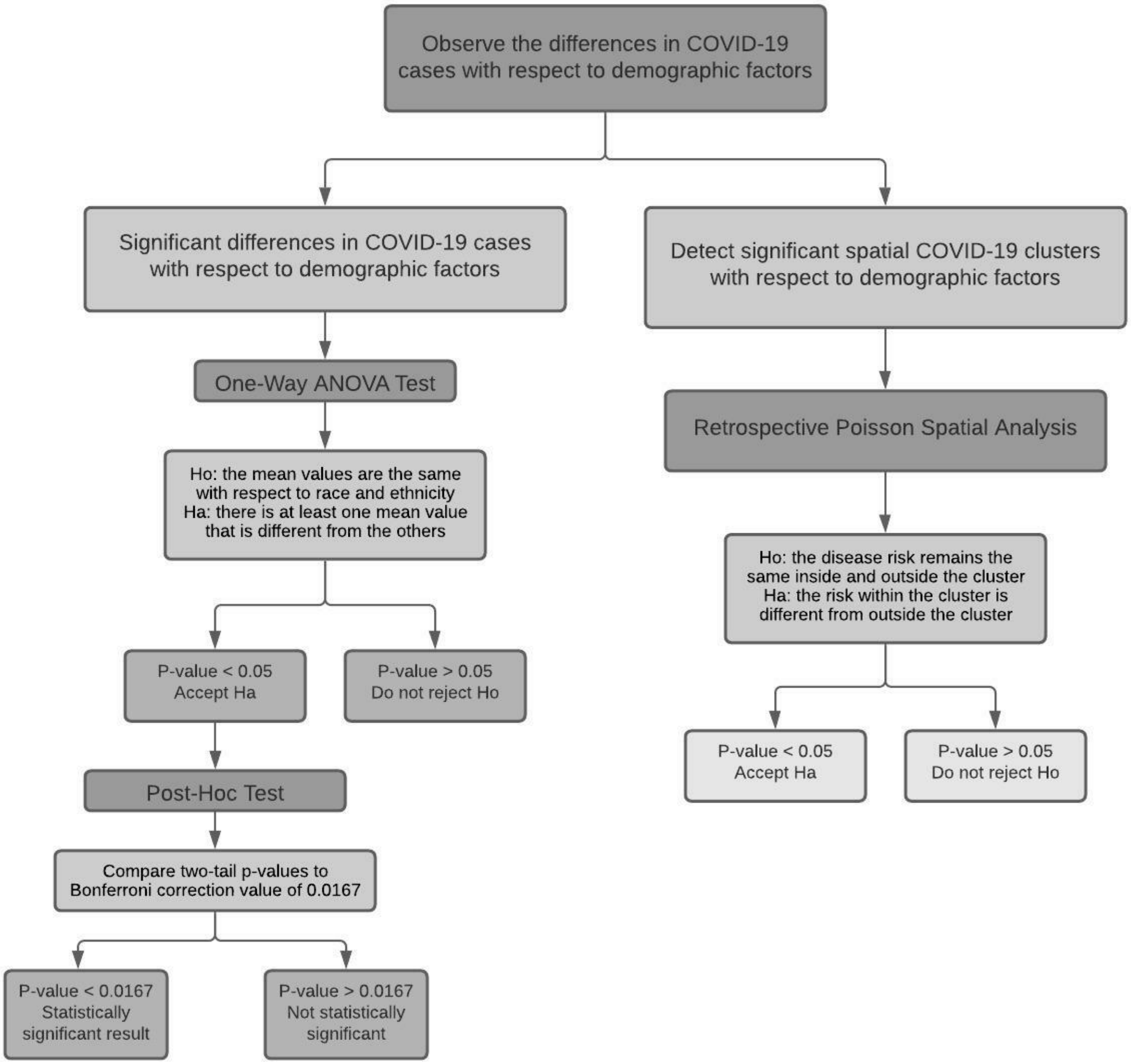

Figure S1: Describing the relations of the selected methods
